# Therapeutic potential of WKYMVm in diseases

**DOI:** 10.3389/fphar.2022.986963

**Published:** 2022-09-02

**Authors:** Huan Ma, Xiaoming Guo, Zhiguo Wang, Mei Han, Hui Liu

**Affiliations:** ^1^ Department of Gastroenterology, Second Hospital of Dalian Medical University, Dalian, Liaoning, China; ^2^ Department of Endoscopy, Second Hospital of Dalian Medical University, Dalian, Liaoning, China

**Keywords:** WKYMVm, FPRs, inflammation, cancer, angiogenesis

## Abstract

The synthetic hexapeptide WKYMVm, screened from a synthetic peptide library, has been identified as an agonist of FPRs with the strongest activating effect on FPR2. WKYMVm plays an anti-inflammatory role in most inflammatory diseases by increasing the chemotaxis of phagocytes and regulating the secretion of inflammatory factors. WKYMVm can inhibit or promote the progression of different types of tumors, which depends on the regulation of WKYMVm on various components such as immune cells, inflammatory factors, chemokines, and tumor epithelial cells. Another major function of WKYMVm is to promote angiogenesis, which is reflected in its therapeutic value in ischemic diseases, wound healing and bone repair. In addition to the above functions, this paper also reviews the effects of WKYMVm on fibrosis, insulin resistance, osteolytic diseases and neurodegenerative diseases. By summarizing related studies, this review can increase people’s comprehensive understanding of WKYMVm, promote its broad and in-depth research, and help to exert its therapeutic value as soon as possible.

## Introduction

Formyl peptide receptors (FPRs) belong to the G protein-coupled receptor family (Le et al., 2001; Ye et al., 2009). Currently, three FPRs have been identified in humans, including FPR1, FPR2, and FPR3 (Ye et al., 2009). Fpr1 and Fpr2 have been found in mice corresponding to FPR1 and FPR2 in humans, respectively (Gao et al., 1998). However, no sequence homologous to human FPR3 has been found in mice. FPRs share significant sequence homology and are encoded by clustered genes, however, they are characterized by different cellular distributions, biological functions and ligand properties (Ye et al., 2009). FPR1 and FPR2 are mainly expressed on monocytes and neutrophils (Le et al., 2001; Ye et al., 2009), but FPR2 is found in a wider range of non-myeloid cells than FPR1, such as endothelial cells, fibroblasts, and nerve cells (Rotrosen et al., 1987; Le et al., 2000; VanCompernolle et al., 2003). FPR3 is expressed in monocytes but not neutrophils (Ye et al., 2009). Both FPR1 and FPR2 have been shown to play critical roles in the progression of a variety of diseases, mainly including cellular chemotaxis under the action of agonists (Krepel and Wang, 2019). They can regulate inflammatory responses, but may have different roles in tumor progression. For example, FPR1 exerts a tumor suppressor function in gastric cancer (GC) by inhibiting angiogenesis (Prevete et al., 2005), while FPR2 has been reported to contribute to tumor progression by promoting invasion and metastasis of GC cells (Hou et al., 2017). Little is currently known about the biological function of FPR3, and few studies have been conducted to elucidate its role (Krepel and Wang, 2019). FPRs can be activated by endogenous or exogenous ligands (Schiffmann et al., 1975; Baek et al., 1996). Due to the high similarity of FPR1 and FPR2, they can share several agonists, including WKYMVm peptides and non-peptide molecules compound 43 and Resolvin D. But different ligands have different affinities for FPRs, for example, the tripeptide fMLF (N-formyl-Met-Leu-Phe) has a high affinity for FPR1, but a low affinity for FPR2. The ligands for FPR2 are more extensive, including polypeptides derived from bacteria and viruses (such as phenol-soluble modulins of Staphylococcus aureus, human immunodeficiency virus (HIV) capsid protein fragments), peptides and lipids produced by the body itself (such as serum amyloid peptide A, antimicrobial peptide LL-37, annexin A1 (ANXA1)), peptides and compounds screened from peptide libraries and compound libraries (such as WKYMVm, mitogen-activated protein kinase 1). FPR3 has only a few high-affinity endogenous ligands, including F2L, acetylated amino-terminal peptide of a heme-binding protein (He et al., 2013; Krepel and Wang, 2019).

WKYMVm (Trp-Lys-Tyr-Met-Val-D-Met or Trp-Lys-Tyr-Met-Val-D-Met-NH2), a synthetic hexapeptide screened from a synthetic peptide library can bind to the FPRs family. WKYMVm has a strong affinity for FPR2, but a weaker affinity for FPR1 and FPR3, so it is considered to be a strong agonist of FPR2 (Baek et al., 1996). Acting on FPRs, WKYMVm can activate phosphatidylinositol 3-kinase (PI3K)/Akt pathway (Zhang et al., 2017), IL-6/gp130 (glycoprotein 130)/signal transducer and activator of transcription 3 (STAT3) signaling pathway (Jun et al., 2021), trigger phosphorylation of intracellular signaling molecules such as extracellular regulated protein kinases (ERK), protein kinase C (PKC), p38 mitogen-activated protein kinase (p38 MAPK), phospholipase C (PLC) and non-signaling proteins such as p47phox (Bae et al., 1999; Ammendola et al., 2004; Cattaneo et al., 2011; Kim et al., 2019). WKYMVm activates the function of neutrophils and monocytes, including chemotaxis, cytokine release, NADPH (Nicotinamide Adenine Dinucleotide Phosphate Hydrogen) oxidase activation, and superoxide production to increase bactericidal activity, which makes it therapeutic against bacterial infections (Baek et al., 1996; Seo et al., 1997; Bae et al., 1999). With the in-depth studies of WKYMVm and FPRs, it has been found that they can not only regulate immune cells and inflammatory factors, but also participate in regulating the function of tissue cells, which makes them also play important roles in tumors, ischemic diseases, fibrosis, insulin resistance, osteolytic diseases and neurodegenerative diseases. The discovery of these effects makes FPRs potential targets for the treatment of various diseases, and also makes WKYMVm a drug with potential therapeutic value. This article reviews the published literature and summarizes the biological functions and regulatory mechanisms of WKYMVm in diseases.

## WKYMVm and diseases

### WKYMVm and inflammation

Inflammation is the body’s complex defense response to trauma, infection, ischemia, toxicity and autoimmune injury (Nathan, 2002; Kuprash and Nedospasov, 2016). The inflammatory process usually leads to recovery and healing after infection, but if not controlled properly, immune cells such as leukocytes and lymphocytes can cause persistent tissue damage, further lead to post-inflammatory fibrosis and promote tumor transformation (Nathan, 2002). The specific receptors of WKYMVm are widely expressed on monocytes, granulocytes, and B lymphocytes, which play a key role in inflammatory response (Seo et al., 1998). Since WKYMVm can promote chemotaxis and inhibit apoptosis of phagocytes, more and more studies have shown that WKYMVm exerts therapeutic effects by inhibiting inflammatory response.

The role of WKYMVm in resisting inflammation caused by bacterial infection is reflected in several aspects. First of all, WKYMVm can enhance the clearance of bacteria. By activating Fpr2, WKYMVm promotes PLC activity, stimulates emergency granulopoiesis, and significantly increases the number of neutrophils in septic mice (Kim et al., 2018). Moreover, WKYMVm can increase hydrogen peroxide (H2O2) production, nitric oxide (NO) release, myeloperoxidase (MPO) and superoxide dismutase (SOD) activities in neutrophils to enhance their bactericidal effects (Kim et al., 2010; Lee et al., 2020). The mechanism involved may be that WKYMVm increases the level of interferon regulatory factor 1 (IRF1) and the phosphorylation of STAT1 at S727, thereby increasing the release of interferon gamma (IFN-γ) in neutrophils (Lee et al., 2020). WKYMVm can also stimulate phospholipase D (PLD) activity through phosphoinositide (PI)-specific PLC and PKC, promote intracellular calcium mobilization, induce the formation of superoxide anion, thereby promoting the killing of *S. aureus* in human monocytes (Bae et al., 1999). In addition, WKYMVm inhibits immune cell apoptosis and improves immune function by inhibiting the activation of caspase-3 (Kim et al., 2010). Second, WKYMVm can regulate inflammatory cytokines. Interleukin (IL)-17 is a pro-inflammatory cytokine, but WKYMVm inhibits the production of pro-inflammatory cytokines (tumor necrosis factor alpha (TNF-α), IL-1β, and IL-6) and upregulates the levels of anti-inflammatory cytokines (IL-10 and transforming growth factor β (TGF-β)) by upregulating IL-17 level, thus exerting anti-inflammatory effects (Kim et al., 2010). Third, WKYMVm can modulate vascular responses under inflammatory conditions. By activating Fpr2, WKYMVm reduces NO production in the aorta of septic mice, reverses lipopolysaccharide (LPS)-induced vascular hyporeactivity, and restores responsiveness to vasoconstrictors (Horewicz et al., 2015). WKYMVm improves the survival rate of mice with sepsis and acute lung injury through the above-mentioned effects. In bronchopulmonary dysplasia (BPD), WKYMVm can upregulate the phosphorylation levels of ERK, and also indirectly upregulate the levels of vascular endothelial growth factor (VEGF) and hepatocyte growth factor (HGF), thereby reducing hyperoxia induced lung inflammation and lung injury, so it may become a new treatment for BPD (Kim et al., 2019). However, for different viral infections, WKYMVm may play different roles. During HIV-1 infection, WKYMVm desensitizes HIV-1 coreceptors C-C motif chemokine receptor 5 (CCR5) and C-X-C motif chemokine receptor 4 (CXCR4) on the surface of monocytes by activating FPR2, thereby effectively inhibiting HIV-1 Env-mediated fusion and viral infection. Furthermore, WKYMVm inhibits the infection of human peripheral monocyte-derived macrophages and CD4^+^ T lymphocytes by R5 or X4 strains of HIV-1, respectively (Li et al., 2001). During influenza A virus (IAV) infection, human alveolar cells expressing FPR2 can be activated by IAV *in vitro*, because the envelope of IAV contains the ligand for FPR2-ANXA1, leading to the increase of viral replication. *In vivo*, WKYMVm increases viral replication and inflammation, and decreases survival of mice after IAV infection (Tcherniuk et al., 2016).

**FIGURE 1:**
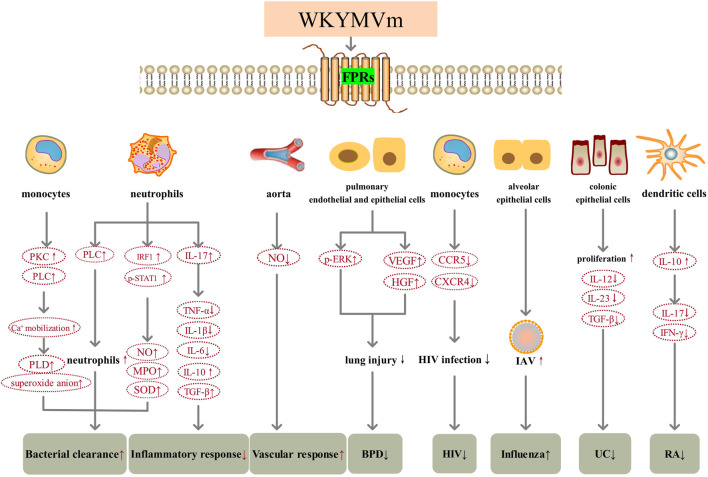
WKYMVm and inflammation. WKYMVm increases bacterial clearance by regulating the number of immune cells in the lesion and increasing various reactive oxygen species production. WKYMVm decreases inflammatory response by modulating inflammatory cytokines. WKYMVm reverses vascular hyporeactivity by reducing NO production in the aorta of septic mice. WKYMVm upregulates the levels of ERK, VEGF and HGF, reduces lung injury in BPD. WKYMVm inhibits the function of CCR5 and CXCR4 on the surface of monocytes and reduces HIV infection. WKYMVm increases IAV replication and aggravates influenza. WKYMVm stimulates colonic epithelial cells proliferation, reduces intestinal permeability, and downregulates inflammatory cytokines expression in UC. WKYMVm stimulates DCs to produce IL-10, decreases the production of IL-17 and IFN-γ, and alleviates cartilage destruction in RA. VEGF, vascular endothelial growth factor; HGF, hepatocyte growth factor; p, phosphorylation; ERK, extracellular signal-regulated kinase; BPD, bronchopulmonary dysplasia; CCR5, C-C motif chemokine receptor 5; CXCR4, C-X-C motif chemokine receptor 4; HIV, human immunodeficiency virus; IL, interleukin; TGF-β, transforming growth factor β; UC, ulcerative colitis; IAV, influenza A virus; NO, nitric oxide; PKC, protein kinase C; IRF1, interferon regulatory factor1; STAT, signal transducer and activator of transcription; IFN-γ, interferon gamma; MPO, myeloperoxidase; SOD, superoxide dismutase; PLC, phospholipase C; PLD, phospholipase D; TNF-a, tumor necrosis factor alpha; RA, rheumatoid arthritis. 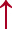, increase; 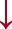, decrease; ↑, aggravate; ↓, alleviate.

WKYMVm also has therapeutic effect on aseptic inflammation. FPR2 has been found to play a key role in regulating colonic homeostasis, inflammation, and epithelial repair (Chen et al., 2013). Fpr2 deficiency can increase the colonic susceptibility to chronic inflammation-related tumors in mice (Chen et al., 2013). In dextran sulfate sodium (DSS)-induced ulcerative colitis (UC) mice, WKYMVm acts on Fpr2 and stimulates colonic epithelial cells proliferation, reduces intestinal permeability, downregulates the expression of inflammatory cytokines (IL-12, IL-23 and TGF-β), reverses intestinal mucosal destruction and colon shortening, and improves clinical scores (including body weight, bleeding, stool) (Kim et al., 2013). In rheumatoid arthritis (RA), WKYMVm stimulates IL-10 production by acting on FPR1 which is expressed on the surface of mature dendritic cells (DCs). Subsequently, IL-10 suppresses Th1 and Th17 cell differentiation, decreases the production of IL-17 and IFN-γ (Park et al., 2021). WKYMVm treatment alleviates cartilage destruction and decreases immune cell infiltration, which may become an immunomodulatory drug to control RA (Park et al., 2021) (Figure 1).

### WKYMVm and cancer

**FIGURE 2:**
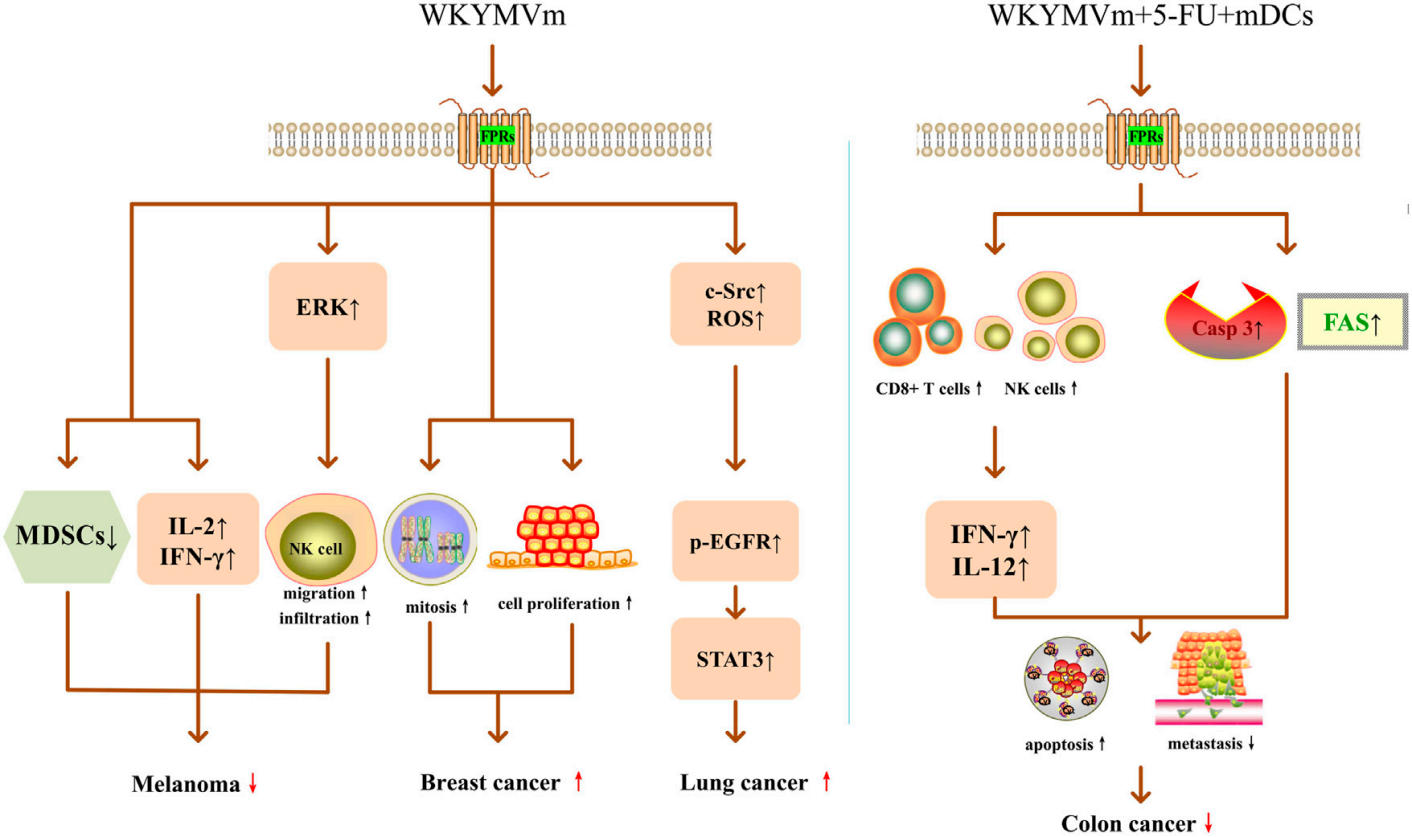
WKYMVm and cancer. Acting on FPRs, WKYMVm reduces the number of MDSCs, upregulates IL-2 and IFN-γ, and promotes NK cell migration and infiltration by activating ERK, thereby inhibiting the growth of melanoma cells. WKYMVm promotes mitosis and proliferation of breast cancer epithelial cells. WKYMVm increases c-Src expression, ROS production, and EGFR phosphorylation, triggers the STAT3 pathway, and promotes lung cancer cell proliferation. Combining with 5-FU and mature DCs, WKYMVm recruits CD8^+^ T lymphocytes and NK cells into tumors to induce the production of IFN-γ and IL-12, increases the expression levels of FAS and caspase-3, thereby inducing apoptosis and inhibiting metastasis of colon cancer cells. ERK, extracellular signal-regulated kinase; IL-2, interleukin-2; IFN-γ, interferon gamma; MDSCs, myeloid-derived suppressor cells; NK, natural killer; casp3, caspase-3; IL-12, interleukin-12; ROS, radical oxygen species; p, phosphorylation; EGFR, epidermal growth factor receptor; STAT3, signal transducer and activator of transcription 3; 5-FU, 5-fluoro-uracil; FAS, Fas cell surface death receptor; mDCs, mature dendritic cells. ↑, increase; ↓, decrease; 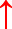, promote; 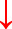, inhibit.

Cancer is one of the leading causes of death in the world, and the morbidity and mortality are increasing year by year (Wang et al., 2018; Lewandowska et al., 2021). In recent years, the immune microenvironment of tumors has become a research hotspot, and many immunotherapy regimens developed based on these studies have played an important role in the treatment of tumors (Wang et al., 2018). Since WKYMVm has regulatory effects on immune cells, its role in tumors has also been proposed and studied. Some researchers suggest that WKYMVm may become a new method to treat tumors. In the mouse model of melanoma, WKYMVm can act on FPRs, reduce the number of myeloid-derived suppressor cells (MDSCs) in tumor tissues, upregulate IL-2 and IFN-γ, promote NK cell migration and increase NK cells infiltration by activating ERK, thereby inhibiting the growth of melanoma cells (Liu J. et al., 2014) (Figure 2). In liver cancer, the FPR2 ligand lipoxin A4 (LXA4) can reduce VEGF secretion and inhibit tumor angiogenesis (Chen et al., 2010), but the role of WKYMVm in liver cancer has not been reported yet. In addition, in patients with solid tumors (GC, non-small cell lung cancer and pancreatic cancer) and acute leukemia, chemotherapy drug treatment leads to neutropenia, but WKYMVm treatment can enhance the bactericidal activity of neutrophils, thereby increasing the resistance to bacteria in tumor patients (Kim et al., 2006; Kim et al., 2008). Due to the limited sample size, this effect of WKYMVm needs to be confirmed by larger clinical studies.

WKYMVm may worsen some tumors. Khau et al. found that ANXA1/FPR2 promoted mitosis of breast cancer epithelial cells by increasing cyclin D1 level, and WKYMVm promoted mitosis and proliferation of breast cancer epithelial cells by activating FPR2 (Khau et al., 2011) (Figure 2). FPR2 can stimulate epithelial ovarian cancer (EOC) to secrete Th2 cytokines through ras homolog family member A (RhoA), increase the polarization of M2 macrophages, and promote the invasion and metastasis of ovarian cancer cells (Xie et al., 2021). Overexpression of FPR2 indicates poor prognosis in EOC patients, and it may be an independent risk factor for EOC prognosis (Xie et al., 2017). In GC, the expression level of FPR2 is positively correlated with the depth of invasion and lymph node metastasis, and negatively correlated with the overall survival rate of patients. FPR2 induces epithelial mesenchymal transformation (EMT) by activating the mitogen-activated protein kinase (MAPK)/ERK pathway, thereby enhancing the invasive and metastatic ability of GC cells (Hou et al., 2017). In glioblastoma, FPR1 promotes VEGF production, induces migration and angiogenesis of human vascular endothelial cells, and accelerates the growth of human gliomas (Zhou et al., 2005). However, whether the treatment of WKYMVm will exacerbate the adverse effects of FPRs on EOC, GC and glioblastoma has not been reported yet.

Some research on the role of FPR2 and WKYMVm in tumors have conflicting conclusions. In lung cancer, Fpr2 can inhibit tumor growth in mice subcutaneously transplanted with Lewis lung cancer (LLC) cells by limiting the macrophage response to the tumor-derived chemokine C-C motif chemokine ligand 2 (CCL2) and inhibiting the polarization of macrophage M2 (Liu et al., 2013). However, as an FPR2 agonist, WKYMVm stimulates FPR2 on the surface of CaLu-6 lung cancer cells, induces NADPH-dependent radical oxygen species (ROS) generation and c-Src activation, promotes epidermal growth factor receptor (EGFR) tyrosine phosphorylation, and transactivation of EGFR, further triggers the STAT3 pathway, thereby promoting lung cancer cell proliferation (Cattaneo et al., 2011) (Figure 2). FPR2 is highly expressed in human colon cancer cell lines, as well as in advanced colon cancer patients. FPR2 promotes proliferation, EMT, angiogenesis and anti-apoptosis of colon cancer cells, and accelerates colon cancer progression (Xiang et al., 2016; Lu et al., 2019). However, Kim et al. found that WKYMVm can act as an adjuvant of anti-tumor drugs to suppress colon cancer progression and metastasis. When WKYMVm combined with a well-known anti-tumor drug-5-fluoro-uracil (5-FU) or combined with 5-FU and mature DCs, the therapeutic effect was superior to single application of 5-FU and double application of 5-FU and mDCs, respectively. Among them, triple therapy with WKYMVm, 5-FU and mature DCs exerted the strongest anti-tumor effect. Triple therapy not only recruits CD8^+^ T lymphocytes and NK cells into tumors to induce the production of IFN-γ and IL-12, but also increases the expression levels of Fas cell surface death receptor (FAS) and caspase-3, thereby inducing apoptosis of colon cancer cells, inhibiting colon cancer cell metastasis, effectively exerting anti-tumor activity and improving survival (Kim et al., 2012) (Figure 2). These contradictory conclusions on FPR2 and WKYMVm in lung cancer and colon cancer need further verification. In general, whether WKYMVm inhibits or accelerates tumor progression depends on different tumor types. Due to the limited number of current studies, more evidence is required to confirm whether WKYMVm can be used as an exogenous anti-tumor drug.

### WKYMVm with angiogenesis and tissue repair

#### WKYMVm and ischemic diseases

**FIGURE 3:**
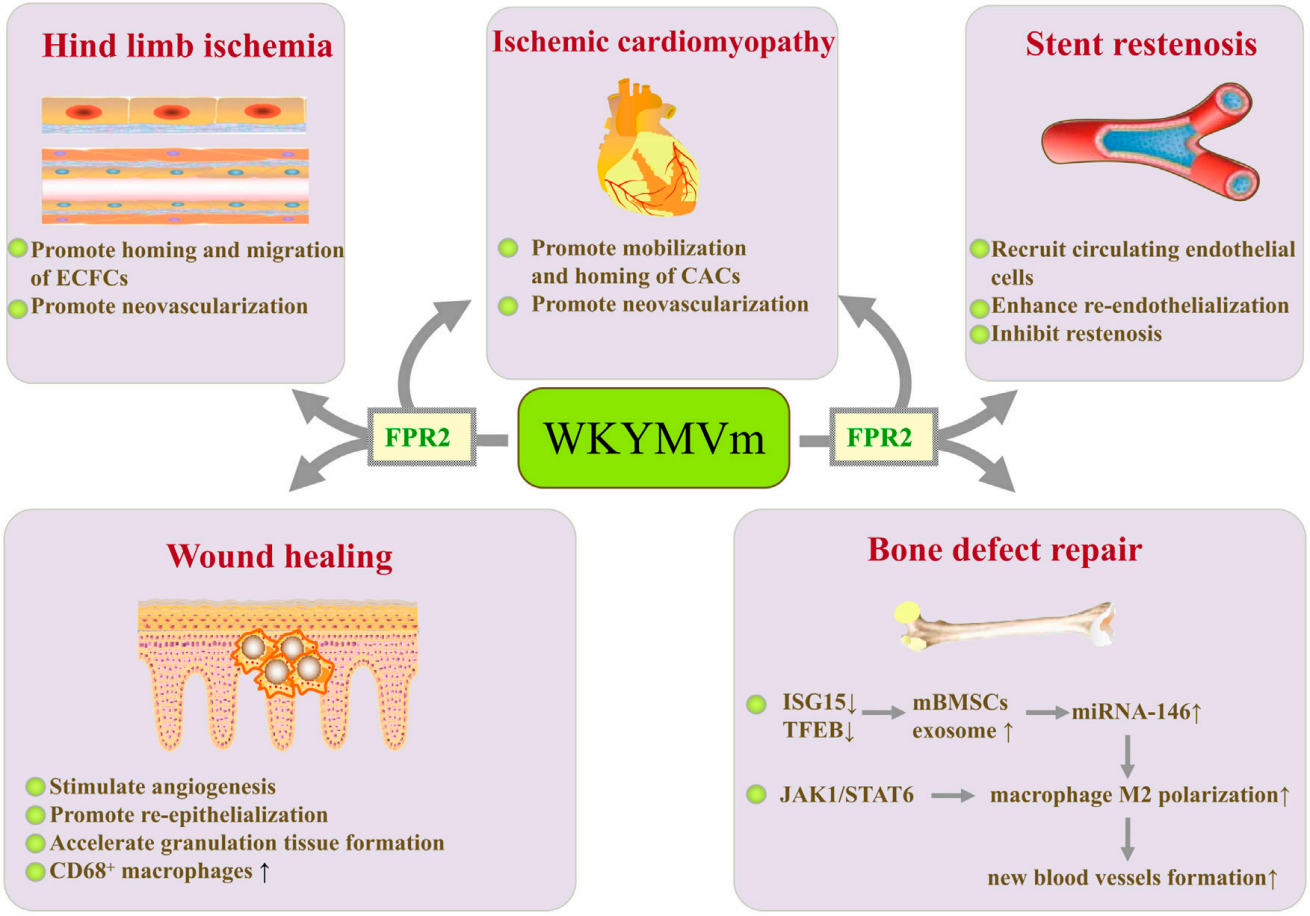
WKYMVm with angiogenesis and tissue repair. WKYMVm promotes homing and chemotactic migration of ECFCs, thereby accelerating neovascularization in ischemic limbs. WKYMVm promotes mobilization and homing of bone marrow CACs to the ischemic heart, which is conducive to neovascularization. WKYMVm and sirolimus continuously coated bare metal stents recruits circulating endothelial cells, enhances re-endothelialization, and inhibits restenosis. WKYMVm stimulates angiogenesis, promotes re-epithelialization, accelerates granulation tissue formation, and increases the CD68^+^ macrophages infiltration in early phase during wound healing. WKYMVm reduces ISG15 and TFEB expression, increases secretion of exosomes containing miRNA-146 in mBMSCs, and promotes the polarization of M2 macrophages. M2 macrophages enhance the formation of new blood vessels to promote bone defect repair. In addition, WKYMVm polarizes macrophages into M2 phenotype by activating JAK1/STAT6 signaling pathway. ECFCs, endothelial colony-forming cells; CACs, circulating angiogenic cells; ISG15, interferon stimulated gene 15; TFEB, transcription factor EB; mBMSCs, murine bone marrow-derived mesenchymal stem cells; JAK1, Janus kinase 1; STAT6, signal transducer and activator of transcription 6. ↑, increase; ↓, decrease.

Intramuscular injection of WKYMVm increases blood perfusion and neovascularization, and reduces tissue necrosis in a mouse model of hind limb ischemia (Heo et al., 2014). By activating FPR2, WKYMVm can promote chemotaxis, endothelial tube formation, and proliferation of human endothelial colony-forming cells (ECFCs) (Heo et al., 2014) and human umbilical vein endothelial cells (HUVECs) (Lee et al., 2006). ECFCs have typical endothelial phenotypes and highly proliferative characteristics, which facilitate them to form new capillaries by differentiating into endothelial cells (Ingram et al., 2005). WKYMVm-FPR2 axis exerts positive effects on homing and chemotactic migration of exogenously transplanted ECFCs into ischemic limbs, thereby promoting neovascularization and improving the therapeutic efficiency of ECFCs in ischemic diseases. In order to overcome the problem of short half-life, Choi et al. fabricated injectable poly (lactide-co-glycolide) (PLGA) microspheres encapsulating WKYMVm peptides for controlled release. Its curative effect was as potent as frequent injection of WKYMVm peptides in restoring blood flow and promoting capillary growth in murine ischemic hind limbs (Choi et al., 2015). This study promotes the possibility of clinical application of WKYMVm. Based on the promoting effect of WKYMVm on homing and neovascularization of ECFCs, several studies have evaluated its role in ischemic cardiomyopathy. By activating Fpr2, WKYMVm promotes the mobilization, homing and neovascularization of bone marrow circulating angiogenic cells (CACs) to the ischemic heart after myocardial infarction, thereby protecting myocardial tissue and function (Heo et al., 2017). However, these are not all effects that WKYMVm exerts on the heart. Jang et al. developed a dual-drug (WKYMVm and sirolimus) coated stent. Their study showed that WKYMVm coating promoted endothelial healing, and WKYMVm and sirolimus continuously coated bare metal stents recruited circulating endothelial cells, enhanced re-endothelialization, and inhibited restenosis (Jang et al., 2015) (Figure 3).

#### WKYMVm and wound healing

It has been found that Fpr1 and Fpr2 are involved in skin wound healing (Liu M. Y. et al., 2014). As agonists of FPRs, WKYMVm has therapeutic effects on diabetic wounds by stimulating angiogenesis, promoting re-epithelialization, and accelerating granulation tissue formation (Kwon et al., 2015). In addition, WKYMVm treatment dramatically increases the CD68^+^ macrophages infiltration in early phase during wound healing, suggesting that WKYMVm can enhance monocytes/macrophages recruitment to the dermis at the early stage of skin wound recovery in diabetic patients (Kwon et al., 2015) (Figure 3). Therefore, the treatment of diabetic wounds by WKYMVm may be achieved not only by stimulating angiogenesis, but also by regulating immune cells. In addition, human lung epithelial cells express functional FPRs, which can respond to endogenous mitochondrial danger signals and promote lung epithelial wound closure (Shao et al., 2011). Fpr2 promotes monocyte migration to mucosal injury sites through the CCL20/CCR6 signaling axis and accelerates intestinal mucosal wound repair (Birkl et al., 2019). By activating Fpr2, helicobacter pylori (2–20) upregulates the expression and secretion of VEGF, promotes the migration and proliferation of gastric epithelial cells, and accelerates the healing of gastric mucosa in rats after indomethacin injury (Paulis et al., 2009). However, whether WKYMVm can activate FPRs to promote pulmonary epithelial repair and gastrointestinal mucosal healing still needs further research.

#### WKYMVm and bone defects

By activating FPR2, WKYMVm reduces interferon stimulated gene 15 (ISG15) and transcription factor EB (TFEB) expression, decreases lysosomal activity, and increases secretion of exosomes in murine bone marrow-derived mesenchymal stem cells (mBMSCs). miRNA-146 in exosomes secreted by mBMSCs promotes the polarization of M2 macrophages (Zhao et al., 2021). M2 macrophages promote bone defect repair through enhancing the formation of new blood vessels (Han et al., 2022). WKYMVm has been reported to polarize macrophages into M2 phenotype by activating Janus kinase 1 (JAK1)/STAT6 signaling pathway, and also plays an important role in increasing platelet-derived growth factor-BB (PDGF-BB) production of M2 macrophages and promoting angiogenesis (Han et al., 2022). These studies provide promising therapeutic strategies for vasculogenesis in bone defect repair (Figure 3).

### WKYMVm and fibrosis

**FIGURE 4:**
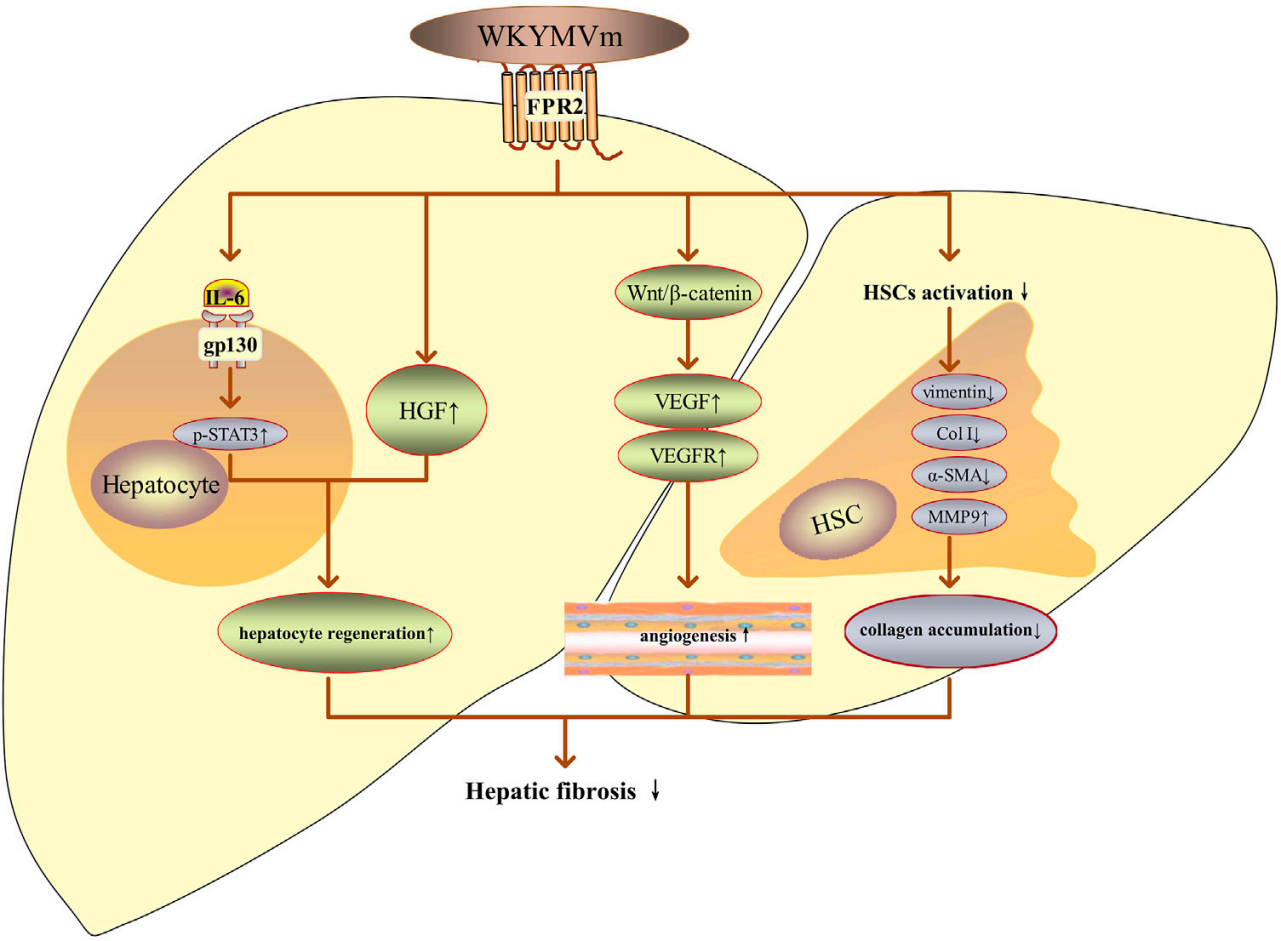
WKYMVm and hepatic fibrosis. WKYMVm improves hepatic fibrosis by decreasing the collagen accumulation, promoting angiogenesis and hepatocyte regeneration. IL-6, interleukin-6; gp130, glycoprotein 130; p, phosphorylation; STAT3, signal transducer and activator of transcription 3; HGF, hepatocyte growth factor; VEGF, vascular endothelial growth factor; VEGFR, vascular endothelial growth factor receptor; HSC, hepatic stellate cell; Col I, type I collagen; α-SMA, alpha-smooth muscle actin; MMP9, matrix metalloproteinase 9. ↑, increase; ↓, decrease.

The anti-fibrotic effect of WKYMVm has been studied in hepatic fibrosis and systemic sclerosis (SSc). WKYMVm or combined with placenta-derived mesenchymal stem cells (PD-MSCs) treatment can inhibit the activation of hepatic stellate cells (HSCs), downregulate the expression of vimentin, type I collagen (Col I) and alpha-smooth muscle actin (α-SMA), increase matrix metalloproteinases (MMPs) activity to decrease the collagen accumulation and alleviate hepatic fibrosis (Jun et al., 2021; Jun et al., 2022). WKYMVm or combined with PD-MSCs also can promote vascular regeneration by upregulating the expression of VEGF and its receptors through the Wnt/β-catenin signaling pathway, improve hepatic regeneration through inducing the release of HGF and activating IL-6/gp130/STAT3 signaling pathway (Jun et al., 2021; Jun et al., 2022). In conclusion, WKYMVm exerts pro-angiogenic, hepatocyte regenerative and anti-fibrotic effects in hepatic fibrosis by activating FPR2 (Figure 4).

However, the current research results on the effect of WKYMVm in SSc are contradictory. Rossi et al. found that the expression of FPRs were increased in dermal fibroblasts from patients with SSc (Rossi et al., 2015; Rossi and Montuori, 2015). By activating FPRs which interacting with the urokinase-type plasminogen activator (uPA) receptor (uPAR), WKYMVm increases proliferation and migration of normal fibroblasts and promotes differentiation of fibroblasts to myofibroblasts, which resulting in increased matrix deposition, αvβ5 integrin and α-SMA expression and ROS generation. So, the activation of FPR2 plays a deteriorating effect in scleroderma (Rossi et al., 2015; Rossi and Montuori, 2015). However, Park et al. reported that WKYMVm decreased dermal thickness and inhibited fibrosis in bleomycin-induced scleroderma mice. They found that WKYMVm inhibited fibroblasts activation, macrophage infiltration, M2-type macrophage generation and inflammatory cytokines (TNF-α and IFN-γ) expression (Park et al., 2019). Notably, in this study, WKYMVm only reduced the production of α-SMA-positive myofibroblasts, vimentin, and phospho-SMAD3 (SMAD family member 3), but did not directly affect the differentiation of TGF-β1-induced fibroblasts to α-SMA-positive myofibroblasts (Park et al., 2019). Therefore, whether WKYMVm can become a new anti-fibrotic therapeutic strategy remains to be further evaluated.

### WKYMVm and insulin resistance

**FIGURE 5:**
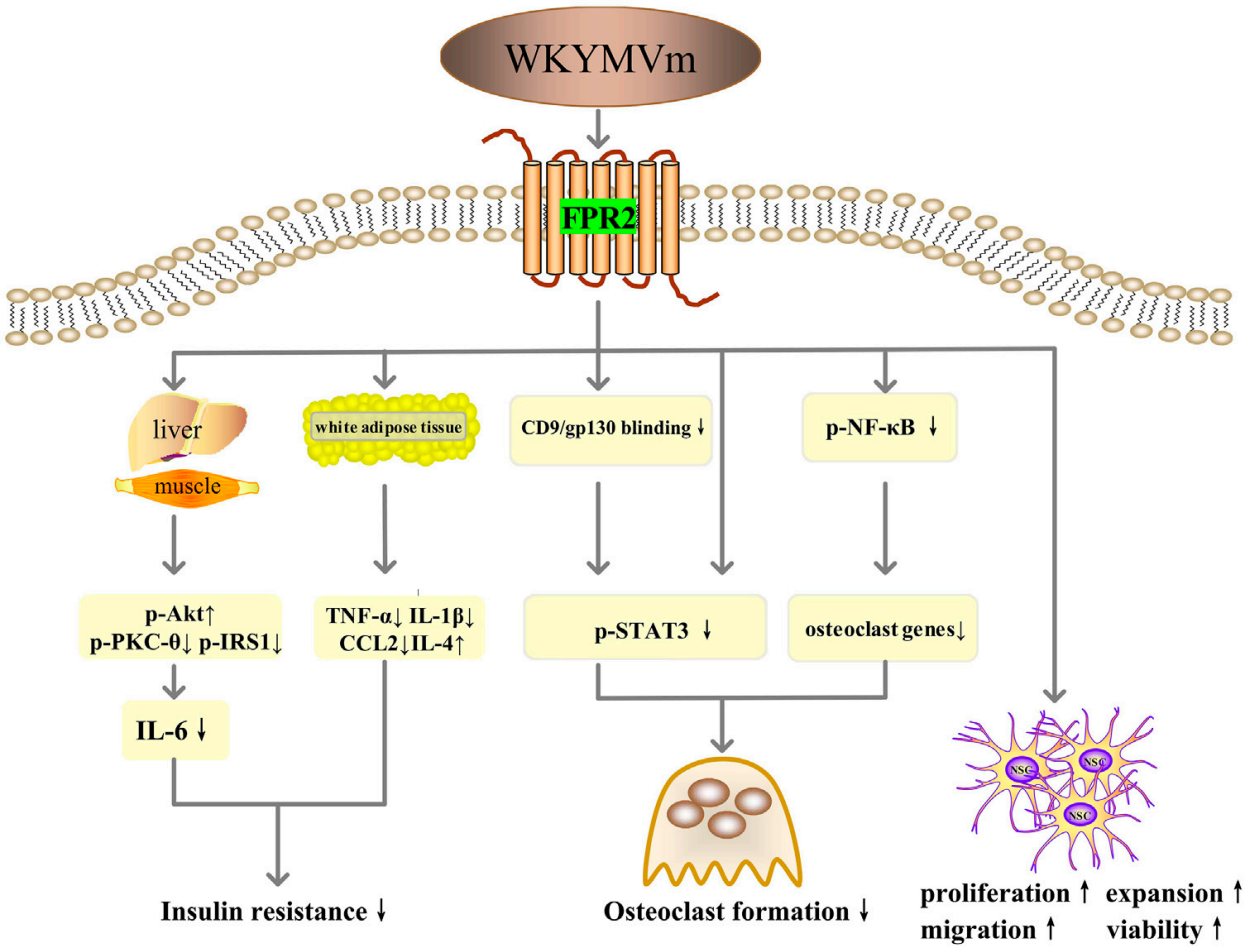
WKYMVm with insulin resistance, osteolytic diseases and neurodegenerative diseases. WKYMVm ameliorates insulin resistance by sensitizing the insulin pathway in metabolic tissues (skeletal muscle and liver), and modulating the expression of inflammatory cytokines in white adipose tissue. WKYMVm alleviates osteolysis by inhibiting the formation of osteoclasts, directly through NF-κB and STAT3 signaling pathway and indirectly through CD9/gp130/STAT3 pathway. WKYMVm promotes the proliferation, expansion, chemotactic migration and viability of NSCs to alleviate neurodegenerative diseases. p-Akt, Akt phosphorylation; p-PKC-θ, protein kinase C-θ phosphorylation; p-IRS1, insulin receptor substrate 1 phosphorylation; IL, interleukin; TNF-α, tumor necrosis factor alpha; CCL2, C-C motif chemokine ligand 2; gp130, glycoprotein 130; p-STAT3, signal transducer and activator of transcription 3 phosphorylation; p-NF-κB, nuclear factor-kappa B phosphorylation; NSC, neural stem cell. ↑, increase; ↓, decrease.

By activating FPR2, WKYMVm can ameliorate insulin resistance by sensitizing the insulin pathway in metabolic tissues (skeletal muscle and liver) of high-fat diet (HFD) mice and palmitate-induced insulin resistance models in L6 myotubes, including increasing insulin-stimulated Akt (S473) phosphorylation, inhibiting the phosphorylation of PKC-θ (T538) and insulin receptor substrate 1 (IRS1) (S307), and reducing IL-6 expression (Yoon et al., 2015). WKYMVm also modulates the expression of inflammatory cytokines in white adipose tissue through downregulating TNF-α, IL-1β, and CCL2 and increasing IL-4 level to improve insulin sensitivity (Yoon et al., 2015). Thus, WKYMVm might be a new therapeutic strategy for insulin resistance (Figure 5).

### WKYMVm and osteolytic diseases

Bone homeostasis depends on the resorption of old bone by osteoclasts and the formation of new bone by osteoblasts (Chen et al., 2018). Overactivity of osteoclasts disrupts this balance, potentially leading to osteolytic diseases such as osteoporosis and inflammatory osteolysis (Chen et al., 2018; Hu et al., 2020). Therefore, inhibiting the formation of osteoclasts may be the key to the treatment of osteolytic diseases. WKYMVm can negatively regulate nuclear factor-κB (NF-κB) ligand receptor activator (RANKL)- and LPS-induced osteoclast differentiation and maturation *in vitro* and alleviate LPS-induced osteolysis in mice models (Hu et al., 2020). The mechanisms involved here include direct regulation through NF-κB and STAT3 signaling pathways and indirect regulation through CD9/gp130/STAT3 signaling pathway (Hu et al., 2020) (Figure 5).

### WKYMVm and neurodegenerative disease

Neural stem cells (NSCs) possess the ability of self-renewal and the potential to differentiate into neurons and glial cells, which makes them possible to have therapeutic value in neurodegenerative diseases such as Alzheimer’s disease, Parkinson’s disease, Huntington’s disease and amyotrophic lateral sclerosis (Gioia et al., 2020). WKYMVm acts on Fpr2 to promote the proliferation, expansion, and chemotactic migration of NSCs, and also enhance the viability of NSCs in adult mice (Kwon et al., 2021). In addition, studies have confirmed that Fpr1 and Fpr2 promote the differentiation of NSCs to neurons through ROS and PI3K/AKT signaling pathways (Wang et al., 2016; Zhang et al., 2017). Therefore, WKYMVm holds promise as a novel approach for the treatment of neurodegenerative diseases (Figure 5).

### WKYMVm and vaccine

DCs are the most important antigen presenting cells (APCs), able to deliver both stimulatory and co-stimulatory signals to T-lymphocytes to trigger their activation and to elicit adaptive immune responses. WKYMVm can promote the expression of CD80 on the surface of mouse bone marrow-derived dendritic cells (mBMDCs) (Lee et al., 2005). When combined with HIV, HBV, and influenza virus DNA vaccines, WKYMVm enhances vaccine-induced CD8^+^ T cells responses in terms of IFN-γ secretion and cytolytic activity in a dose-dependent manner (Lee et al., 2005). These indicate that WKYMVm peptide can be used as a new adjuvant for DNA vaccines.

## Conclusion

As an exogenous agonist, WKYMVm is involved in the regulation of various cells (e.g., neutrophils, monocytes/macrophages, lymphocytes, DCs, NK cells, vascular endothelial cells, HSCs, skin fibroblasts), pro-inflammatory cytokines (e.g.,TNF-α, IL-1β, IL-6), and anti-inflammatory cytokines (e.g., IL-10, TGF-β) after the activation of FPRs. WKYMVm can regulate various functions such as cell proliferation, chemotaxis migration, differentiation, and apoptosis. These effects make WKYMVm play active roles in most inflammatory diseases, ischemic diseases, tissue damage repair, hepatic fibrosis, insulin resistance, osteolytic diseases, and neurodegenerative diseases. WKYMVm may play favorable or unfavorable roles in tumors, which depending on different tumor types. However, there are conflicting results on the role of WKYMVm in lung cancer, colon cancer and SSc. In this paper, studies on WKYMVm are reviewed in detail, in order to provide a more comprehensive understanding of WKYMVm. Through this review, it can be found that in the past researches, there are still deficiencies and contradictions on the role of WKYMVm in some diseases. Therefore, further research in these areas is needed in the future to clarify the role of WKYMVm and accelerate its clinical application.

## Glossary

ANXA1: annexin A1
APCs: antigen presenting cells
BPD: bronchopulmonary dysplasia
CACs: circulating angiogenic cells
CCL2: C-C motif chemokine ligand 2
CCR5: C-C motif chemokine receptor 5
Col I: type I collagen
CXCR4: C-X-C motif chemokine receptor 4
DCs: dendritic cells
DSS: dextran sulfate sodium
ECFCs: endothelial colony-forming cells
EGFR: epidermal growth factor receptor
EMT: epithelial mesenchymal transformation
EOC: epithelial ovarian cancer
ERK: extracellular regulated kinase
FAS: Fas cell surface death receptor
fMLF: N-formyl-Met-Leu-Phe
FPRs: formyl peptide receptors
5-FU: 5-fluoro-uracil
GC: gastric cancer
gp130: glycoprotein 130
HFD: high-fat diet
HGF: hepatocyte growth factor
HIV: human immunodeficiency virus
H2O2: hydrogen peroxide
HSCs: hepatic stellate cells
HUVECs: human umbilical vein endothelial cells
IAV: influenza A virus
IFN-γ: interferon gamma
IL: interleukin
IRF1: interferon regulatory factor 1
IRS1: insulin receptor substrate 1
ISG15: interferon stimulated gene 15
JAK: Janus kinase
LLC: Lewis lung cancer
LPS: lipopolysaccharide
LXA4: lipoxin A4
MAPK: mitogen-activated protein kinase
mBMDCs: mouse bone marrow-derived dendritic cells
mBMSCs: marrow-derived mesenchymal stem cells
MDSCs: myeloid-derived suppressor cells
MMPs: matrix metalloproteinases
MPO: myeloperoxidase
NADPH: Nicotinamide Adenine Dinucleotide Phosphate Hydrogen
NF-κB: nuclear factor-kappa B
NO: nitric oxide
NSCs: neural stem cells
PDGF-BB: platelet-derived growth factor-BB
PD-MSCs: placenta-derived mesenchymal stem cells
PI: phosphoinositide
PI3K: phosphatidylinositol 3-kinase
PKC: protein kinase C
PLC: phospholipase C
PLD: phospholipase D
PLGA: poly (lactide-co-glycolide)
p38MAPK: p38 map kinase
RA: rheumatoid arthritis
RANKL: ligand receptor activator
RhoA: ras homolog family member A
ROS: radical oxygen species
α-SMA: alpha-smooth muscle actin
SMAD3: SMAD family member 3
SOD: superoxide dismutase
SSc: systemic sclerosis
STAT: signal transducer and activator of transcription
TFEB: transcription factor EB
TGF-β: transforming growth factor beta
TNF-α: tumor necrosis factor alpha
UC: ulcerative colitis
uPA: urokinase-type plasminogen activator
uPAR: urokinase-type plasminogen activator receptor
VEGF: vascular endothelial growth factor
